# “We can’t handle things we don’t know about”: perceived neurorehabilitation challenges for Malawian paediatric cerebral malaria survivors

**DOI:** 10.1186/s12887-020-02405-1

**Published:** 2020-11-03

**Authors:** Alexandra Boubour, Sebastian Mboma, Tracy Võ, Gretchen L. Birbeck, Karl B. Seydel, Macpherson Mallewa, Dorothy Chinguo, Melissa Gladstone, Suraya Mohamed, Kiran T. Thakur

**Affiliations:** 1 Department of Neurology, Columbia University Irving Medical Center, New York, NY USA; 2 Blantyre Malaria Project, University of Malawi College of Medicine, Queen Elizabeth Central Hospital, Blantyre, Malawi; 3 School of Public Health, University of the Western Cape, Cape Town, Republic of South Africa; 4 Department of Sociomedical Sciences, Columbia University Mailman School of Public Health, New York, NY USA; 5 Epilepsy Division, Department of Neurology, University of Rochester Medical Center, Rochester, NY USA; 6 Epilepsy Care Team, Chikankata Hospital, Mazabuka, Zambia; 7 College of Osteopathic Medicine, Michigan State University, East Lansing, MI USA; 8 Malawi-Liverpool Wellcome Trust Clinical Research Programme, Blantyre, Malawi; 9 Paediatric Department, University of Malawi College of Medicine, Blantyre, Malawi; 10 Department of Physiotherapy and Occupational Therapy, Queen Elizabeth Central Hospital, Blantyre, Malawi; 11 Department of Women and Children’s Health, Institute of Translational Medicine, University of Liverpool, Liverpool, UK; 12 Division of Critical Care & Hospitalist Neurology, Columbia University Irving Medical Center, New York, NY USA; 13 Columbia University Irving Medical Center and New York Presbyterian Hospital, NY, USA

**Keywords:** Neurorehabilitation, Paediatric, Cerebral malaria, Qualitative, Malawi

## Abstract

**Background:**

We sought to identify perceptions of neurorehabilitation challenges for paediatric cerebral malaria (CM) survivors post-hospital discharge at Queen Elizabeth Central Hospital (QECH) in Blantyre, Malawi.

**Methods:**

An exploratory approach was used to qualitatively investigate the perceived neurorehabilitation challenges for paediatric CM survivors. Data were collected through semi-structured in-depth interviews (IDIs) and focus group discussions (FGDs). Eighteen data-gathering sessions were conducted with 38 total participants, including 3 FGDs with 23 primary caregivers, 11 IDIs with healthcare workers at QECH, and 4 IDIs with community-based rehabilitation workers (CRWs).

**Results:**

FGDs revealed that caregivers lack important knowledge about CM and fear recurrence of CM in their children. Post-CM children and families experience substantial stigma and sociocultural barriers to integrating into their community and accessing neurorehabilitative care. At a community-level, rehabilitation infrastructure, including trained staff, equipment, and programmes, is extremely limited. Rehabilitation services are inequitably accessible, and community-based rehabilitation remains largely unavailable.

**Conclusions:**

There is an urgent need to establish further training of rehabilitation personnel at all levels and to build accessible rehabilitation infrastructure in Malawi for post-CM patients. Additional work is required to expand this study across multiple regions for a holistic understanding of neurorehabilitation needs.

## Background

Cerebral malaria (CM) is characterised by peripheral *Plasmodium falciparum* parasitaemia and an unrousable coma persisting > 30 min post-seizure not attributable to an identifiable alternative cause [1]. As the most severe complication of malaria, CM is a major life-threatening disease, particularly among children aged ≤5 years living in malaria-endemic regions of sub-Saharan Africa [2]. Despite antimalarial treatment, CM has a mortality rate of 15–20% in children [3, 4]. In over one-third of patients, CM is associated with debilitating neurological sequelae, including memory impairment, seizure disorders, paralysis, hyperactivity, speech impairment, behavioural changes, and movement disorders [1, 5–9]. Neurorehabilitation is effective to improve longitudinal prognosis and mitigate neurological sequelae in post-CM children [7, 10, 11].

In resource-limited settings, there is a paucity of neurorehabilitation in practice nor clear guidelines to inform rehabilitation in the post-CM period [11–13]. In Malawi, there is a strong focus placed on the prevention and management of childhood disease and limited infrastructure to address the consequences of these diseases. Innovative studies in India, Brazil, Lesotho, Bangladesh, and Kenya have investigated the design and implementation of a successful community-based neurorehabilitation program in the context of limited resources [11, 14–17]. Further, the use of traditional and computer-assisted neurorehabilitation has been shown to improve baseline attention, memory, and executive functioning in post-CM children in Uganda; however, this may not be feasible in Malawi due to cost and technological limitations in rural regions [11, 12]. Community-based rehabilitation has existed in Malawi since 1987; however, training programmes are remain limited [18]. Existing public neurorehabilitation programmes provide services for little to no cost to patients in Malawi; however, these services are centralised in urban areas and largely inaccessible to patients living outside cities. Due to the lack of funding allocated to these public programmes, most neurorehabilitation infrastructure – including community-based programmes – are exist privately through nongovernmental organizations (NGOs), are costly for those seeking care, and unsustainable given their external funding (through NGOs and charitable organizations rather than through the national health system).

As such, these therapies remain inequitably available, and it is not currently understood what constitutes an effective, comprehensive, and sustainable neurorehabilitation programme for post-CM children [19]. This study sought to identify perceived neurorehabilitation challenges for paediatric CM survivors post-hospital discharge in Blantyre, Malawi, a resource-limited setting.

## Methods

### Study aim and design

In this observational study, an exploratory approach was used to qualitatively investigate the perceived challenges for paediatric CM survivors [20].

### Study setting and population

The study population included primary caregivers of CM survivors admitted to the Paediatric Research Ward (PRW) at Queen Elizabeth Central Hospital (QECH). Caregivers were recruited prior to patient discharge from the PRW, at which point their child (the CM patient) was already enrolled in a quantitative sub-study assessing longitudinal neurosequelae and neurorehabilitation needs. At least one-month post-hospital discharge, caregivers returned to the hospital so that patients could undergo routine follow-up assessments. Data from the quantitative sub-study was used to determine which patients exhibited neurosequelae and required neurorehabilitation at the time of follow-up, and caregivers of these patients were invited to an FGD. No patients died at home; thus, no patients were excluded for this reason.

The study population additionally included healthcare workers at QECH (in-hospital rehabilitation officers, clinical officers, nurses, and physicians who specialise in the care of CM patients) and community-based rehabilitation workers (CRWs) (based at non-governmental organisations (NGOs) or community-based organisations (CBOs)) who work directly with children in community-based rehabilitation teams. QECH is an urban tertiary referral centre in Blantyre, Malawi and one of two hospitals in-country with staff and infrastructure to treat children with brain injury and severe neurological issues [10]. The PRW is a specialised unit with well-trained staff and over 30 years of expertise researching and managing high-risk children with CM.

### Sampling and inclusion criteria

We employed a non-probability purposive sampling technique such that selected participants fit study inclusion criteria (Table 1) [21, 22]. Participants were excluded if they did not fit the criteria specified in Table 1. Sample size was determined by theoretical saturation [23].

**Table 1:** Study Inclusion Criteria

Participant Type	Inclusion Criteria
Caregivers of CM Survivors	• Primary caregiver of child who survived CM • Child must already be enrolled in the COPS study at QECH • Residence in Blantyre at time of enrolment • Aged ≥18 years
Healthcare Workers at QECH	• Employed on the malaria ward at QECH or COPS study • Working for ≥2 years at QECH providing treatment, physiotherapy, and/or occupational therapy to children with CM
CRWs	• Working in rehabilitation for ≥2 years with NGOs or CBOs that provide rehabilitation services within Blantyre District

### Data collection

Data were collected using semi-structured in-depth interviews (IDIs) and focus group discussions (FGDs) [24, 25]. Healthcare workers and CRWs completed IDIs, which explored individual perceptions and experiences of caring for post-CM children, access to rehabilitative services, and existing gaps in infrastructure to care for post-CM children, which enabled free and insightful pursuit of ideas by participants [20]. Caregivers were placed in FGDs, which allowed sharing of personal experiences and perceptions of caring for post-CM children and existing rehabilitation support. We piloted and employed semi-structured guides for the IDIs and FGDs (Appendix). Prior to conducting IDIs and FDGs, interviewers and translators were familiarised with the semi-structured guide. Semi-structured guide piloting was informed by a grounded theory approach, and guides were reviewed and modified through an iterative process as IDIs and FDGs were carried out and new themes emerged. IDIs were conducted in English or the vernacular language, Chichewa, and FGDs were conducted in Chichewa by a male Malawian social scientist (SM).

We completed 18 data-gathering sessions with 38 total participants, including three FGDs with 23 primary caregivers, 11 IDIs with healthcare workers at QECH, and four IDIs with CRWs (Tables 2 and 3). CRWs were identified through the Malawi Council for the Handicapped and were not employed by the Malawian government nor through research projects at QECH. Each FGD comprised of both males and females; 22 primary caregivers were the parents of CM survivors and one caregiver was an elder sister.

**Table 2:** In-depth Interview and Focus Group Demographics. In-Hospital Healthcare Workers and CRWs

Profession	Number of Interviewees
In-Hospital Healthcare Workers (Total)	11
*Rehabilitation Officers*	4
*Clinical Officers*	3
*Nurses*	2
*Physicians*	2
CRWs	4
Total	15

**Table 3:** In-depth Interview and Focus Group Demographics. Focus Groups for Caregivers of CM Survivors

Focus Group	Number of Females	Number of Males	Total
FGD 1	5	3	8
FGD 2	5	4	9
FGD 3	5	1	6
**Total**	**15**	**8**	**23**

### Data management and analysis

IDIs and FGDs were audio recorded and later transcribed verbatim. Interviews completed in Chichewa were transcribed in Chichewa and subsequently translated to English. All transcripts were assessed for quality before analysis. A qualitative researcher (TV) and research assistant (AB) independently conducted manual thematic content analysis on the transcripts [26]. In our approach, we acknowledged that all information provided was the subjective experience of those interviewed and may have been influenced by the researchers. We conducted a de novo line-by-line analysis of each transcript and inductively identified main concepts arising from transcript sections. Next, we highlighted and provided an idea for a code or important defining category for each concept. The team coded 18 transcripts separately and then reviewed the codes before creating a final list of codes for analysis.

### Rigour

We maintained a reflective practice and applied methods triangulation by using both IDIs and FGDs during data collection. We also used data-source triangulation by gathering perspectives from multiple sources, including caregivers, in-hospital healthcare workers, and CRWs [27]. Methods triangulation was applied in analysis [27]. We employed peer-debriefing through continuous discussion regarding any issues with data collection, data analysis, and finding documentation.

### Ethical considerations

Ethics approvals were granted by the Institutional Review Board of Columbia University Irving Medical Center (New York, New York, United States) and Ethics Committees of University of Malawi College of Medicine (Blantyre, Malawi) and University of the Western Cape (Bellville, South Africa). Informed consent was obtained from all enrolled caregivers and healthcare workers. Caregivers were consented on the PRW before their child’s discharge once CM was no longer acute. For caregivers, recruiters explained that participation would not impact the care of their child; for healthcare workers, recruiters explained that participation would not affect their employment. All IDIs and FGDs took place in a private room to maintain participant privacy. Hard-copy data were stored in a secure, locked room to maintain participant confidentiality, and digital data were stored as encrypted files on a password-protected computer. Audio files and transcripts were de-identified.

## Results

Six primary themes were generated through our analysis. An overview of emerging themes and their sources is described in Table 4. Themes, descriptions, and supporting quotes are provided in Table 5. An overview of existing processes and practices surrounding neurorehabilitation at the health facility-level is provided below.

**Table 4:** Emerging Qualitative Themes and Sources

Emerging Themes	Caregivers	In-Hospital Healthcare Workers	CRWs
Gaps in Caregiver Education and Knowledge about CM	✓	✓	✓
Caregiver Fear of CM Recurrence	✓		
Inability to Fund Rehabilitative Infrastructure		✓	✓
Disability Stigma and Sociocultural Barriers to Accessing Rehabilitative Care	✓	✓	✓
Challenges to Continuing Care in the Community	✓	✓	✓
Suggestions for Implementing Community-Based Rehabilitation	✓	✓	✓

**Table 5:** Themes, description, and supporting quotes

Theme	Description	Supporting Quotes
**(1) Gaps in Caregiver Education and Knowledge about CM**	In-hospital healthcare workers reported that caregivers are often discharged with little information about their child’s illness, including its cause and long-term effects. Caregivers of physically disabled children discussed that they do not know how to support their children at home.	“We do not give much information to these guardians. Many guardians would like to know maybe the cause of the condition, what would happen afterward; would there be a chance where the patient will be normal again or not? And if not, if the patient will not be back to normal, what support can they give? I think we need to give the guardian enough information on that issue.” (Clinical Officer 2) “It is important that we are trained in skills on how to take care of the children at home. In short, we should be trained on how to assist the children so that they can be independent.” (Caregiver FGD 1)
	The absence of standardised discharge and follow-up plans for CM patients has resulted in miscommunications between the medical team and patients’ families. A standardised discharge plan might enable caregivers to be better educated about the effects of CM on their children.	“The communication between the medical team and the rehabilitation team – like we said sometimes they discharge patients without our knowledge, if we communicated well we would have a chance to teach the mother and do enough physio with the child so they are discharged while they are doing well.” (Rehabilitation Officer 1) “There is no standard care, there are no educational materials that are given to them. It is not in standard of care to give educational material to read about what to do if XYZ [neurological sequelae] develop.” (Rehabilitation Officer 2)
	CM education could take place in the community rather than in-hospital; the government could assist by educating community health workers about CM so that community health workers can transfer knowledge to caregivers.	“If the government can educate their health workers about the complications and just have them tell the caregiver what they will need to do to help their child, that will also help because those children are dying, and the caregivers don’t know what to do with them.” (Nurse 1)
	Some in-hospital healthcare workers and CRWs reported to have given detailed advice to caregivers of CM survivors regarding childcare post-hospital discharge.	“The moment we have started rehab in the hospital with that child, automatically we start preparing that mother for discharge, so whatever we do we tell them that you must continue to do this at home when you get discharged.” (Rehabilitation Officer 1) “We talk to mother advise them that maybe this child might not be himself or herself again or the way they were before, they might not behave like normal kids because of the effect that malaria has had on their brain, so we do encourage mothers that they should understand and love the kid the way they are.” (Clinical Officer 2)
	Caregivers acknowledged receipt of advice (regarding childcare post-hospital discharge) while at the hospital, described parenting changes that arose as a result of this advice, and described that they have faith and trust in the medical providers treating their children at the hospital.	“When I’m doing the household chores, I try to engage my child in some of them. For instance, I tell her to boil water for her bath. Furthermore, I help her with her homework. Sometimes, she forgets to do her homework, so I have to remind her. In addition, she loses her coordination, so I engage her in light exercises, such as running.” (Caregiver FGD 1) “When the child is getting discharged in the hospital, we get relieved of some of the worries because of the pieces of advice given to us by the doctors on how to take care of the child. We trust the judgment made by the hospital personnel and we are always told to be free to return if the child falls sick again.” (Caregiver FGD 2) “I was advised to let the child play with friends who are not violent and to let her to do simple household chores such as washing plates. I was also told to buy her toys to play with. I could see that the strategy was working after washing two plates, she could ask for more. That gave me hope that the child was recovering.” (Caregiver FGD 2)
**(2) Caregiver Fear of CM Recurrence**	Caregivers expressed worry that their child would never fully recover to how he/she was before falling ill with CM.	“I’m still afraid what the future holds for this child because of the cerebral malaria she suffered from. I still get worried because whenever she has fever.” (Caregiver FGD 2) “My greatest concern was whether she would get well ever again. I didn’t know that she would be able to crawl and play with her friends[…]she has not yet been enrolled in school because there are so many things that she can’t do by herself.”(Caregiver FGD 3)
**(3) Inability to Fund Rehabilitative Infrastructure**	There is a lack of funding to administer free medical equipment, including cerebral palsy (CP) chairs and wheelchairs, to patients in need of it for daily use.	“[…]children with physical disabilities need assistive devices, at first we had some funding to buy assistive device that we were providing such as wheel chairs and corner CP chairs but we no longer have such funding.” (Rehabilitation Officer 1)
	For healthcare staff, limited training exists to specialise in rehabilitation, particularly neurorehabilitation, and there are limited opportunities for work due to a dearth of rehabilitation service infrastructure.	“It’s very hard especially like here in Malawi; you cannot get anywhere. You cannot get that training unless you go outside the country. The expert I was working with was trained in Canada.” (CRW 1) “Physical disabilities have got experts on how to tackle with them. Behaviour problems are different altogether[…]When a child is taken to hospital, often clinician will brush them off saying you know what, just discipline your child, but the problem may not be a simple discipline problem.” (Physician 3)
	The lack of rehabilitation staff creates a barrier to provide adequate care for CM survivors with neurological sequelae.	“The other thing, the availability of staff, there are few of us so it becomes difficult to manage a lot of children at once, you can’t manage you only do minor assessments.” (CRW 3)
	Caregivers expressed difficulty in accessing services centralised in the city due to inability to fund transport to the city hospital.	“Usually you give [caregivers] a date to come [to appointments], but you find that maybe they didn’t come because of issues to do with transport. Most of the times they say they don’t have [money].” (Rehabilitation Officer 2)
**(4) Disability Stigma and Sociocultural Barriers to Accessing Neurorehabilitative Care**	For post-CM patients with disabilities, social isolation presents as a barrier for patients and their families to engage in community activities.	“They are not able to participate in community projects such as food for work, they say such people are too busy taking care of their kids, they can’t go to churches or weddings, and people speak harshly of them.” (Rehabilitation Officer 1)
	Caregivers discussed stigma, including community shame, beliefs about their post-CM child being bewitched, and others mocking the post-CM child.	“I lack peace of mind because even my own relatives used to make fun of me. They believed that the child was bewitched.” (Caregiver FGD 1) “They don’t really understand what has happened to them because even today people think they have been bewitched so if you really don’t understand what happened it is also difficult for you to take care of that child because you don’t know, you just think that possibly the child is having the problem with the hand, you don’t know that the problem may be coming from the brain.” (CRW 1)
	Children with behavioural problems may be forced to drop out of school by school headmasters.	“These children[…]are like street kids, they just go begging because they cannot do anything, they will be like that when they will grow up, doing that and in the end those children will develop a very bad habits because they are not empowered” (Nurse 2).
	Caregivers and healthcare workers expressed that a support group would be beneficial means of social support for caregivers more than post-CM children.	“I wish you could establish small organisation/committees comprising of parents and guardians whose children suffer/suffered from cerebral malaria. These members can help others who may face similar problem. As of now, we just teach/share things that we feel we know, not necessarily skills or knowledge from the hospital.” (Caregiver FGD 2) “[Caregiver support groups are] being shown elsewhere to help a lot[…]Most of these parents there at home are frustrated no-one want listen to them. Exchanging experiences of problems that they are facing sometimes is half solved.” (Physician 3)
**(5) Challenges to Continuing Care in the Community**	Caregivers noted that they do not know of any CBOs providing education and support for families with post-CM children and stated that these organisations should exist.	“[…]There should be the small organisations in the communities to help parents who shall have the children with the same problem, you should train them because we do it ignorantly. Most of the staff we train our children is from our heads without any experience.[…]There should be an organisation made up of people who had the same experience.” (Caregiver FGD 3)
	Care does not often continue in the community following hospital discharge, which poses as a barrier to patient recovery.	“The moment the child has been discharged from the [central hospital] and goes to [their village], there is nothing to be done there.” (CRW 3) “When the child goes home [from the hospital] he or she will no longer receive any rehabilitation as a result the child just stays at home without proper assistance.” (CRW 4)
	Many families, especially those living in rural settings, face long distances to health facilities, and unfavourable modes of transportation for physically disabled children.	“If you ask [caregivers] to come to the clinic with the child, it is not easy, they need transportation they use a minibus and they have to carry their child on their back for some distance as such you cannot expect them to come to clinic as regularly as possible.” (Physician 3)
	Healthcare workers elaborated that, with lacking social support and difficulties accessing existing follow-up care, caregivers can easily become overwhelmed with taking care of their post-CM child. Some caregiver experiences were contradictory and emphasised a lack of support in the community.	“A priority may be to take care of other children they have. They need to take care of the family and their husbands. So, the child with disability is like a burden to them, and they will not take good care of the child because they do not have enough support” (Nurse 1) “Most of these parents would like to go out and look after money may be to go in the fields to farm. So, they would need someone to look after this sick child. So, when they are sitting for this child they are not going out they would lack enough money they would lack food. And this child also would need food to eat.” (Nurse 2) “In my community there are no rehabilitation programmes for children that have neurological problems due to malaria, they take this as a family problem and the community is not concerned at all.” (Caregiver FGD 2) “[…]in the community where I live, people look at the impairment of my child as a family burden or just as any other disease my affect a family and the community has nothing to do about it.” (Caregiver FGD 3)
	This lack of community-based support extends to the school-setting, where there are no teachers nor programmes available to accommodate post-CM children with special needs.	“Most of the teachers are not busy with them, we don’t really have special need teachers so in most of the schools they just see all the children as equal they don’t really see the problems, they just teach them as part of children not that they have brain problems.” (CRW 3)
	Most participants perceived community-based rehabilitation as a critical component in caring for children who have survived CM and subsequently developed neurodisabilities.	“If we can develop rehabilitation centres in the communities so that those kids once they have developed complications, they can be taken care of in those rehabilitations centres. I think that can be a relief to their caregivers who most of their times are busy taking care of these kids.” (Clinical Officer 2) “Yes, it is very important to follow-up children that had cerebral malaria and have been discharged from hospital ward as this helps in early diagnosis and rehabilitation of any problems that may arise before these problems can reach severe stages. It is really important!” (Caregiver FGD 3)
**(6) Suggestions for Implementing Community-Based Rehabilitation**	Providing incentives to caregivers in the form of food vouchers or transport funding may be efficacious to improve follow-up appointment adherence, especially in instances where attending care in the community is not feasible.	“Apart from giving [caregivers] materials, you also need to give them something that will motivate them to attend follow-ups because sometimes the mothers are not able to attend even the community rehabilitation they are busy doing other businesses so you can give them something that will motivate them, such as supplementary foods like chiponde.” (CRW 1)
	Providing a palliative care team with cars to travel to villages for care provision and assessment may mitigate current challenges to providing patients with transportation funds to return to the hospital.	“I think the best thing is that the cerebral malaria group would do emulate what palliative care people are doing, they need to form a group of people which must follow-up these children who have brain damage and see how they are being cared for, are they well at home, what are their problem or how can we help, do they need wheel chairs or do they need CP chairs?” (Nurse 1)
	Healthcare workers were adamant that more experts be trained in neurodisability management to increase the available labour force when scaling up infrastructure of community-based rehabilitation services.	“The gap is starts from training because you cannot have public community rehabilitation programme without data skills in identifying and support those kids.” (Physician 3)
	There is a need to gather epidemiological data on neurological disability following CM to inform the building of rehabilitation infrastructure in Malawi and emphasise the breadth of this public health problem.	“It is only when we [have epidemiological data] that when we can convince government or non-governmental organisations to think about better implementation of community-based rehabilitation programmes.” (Physician 3)

Physiotherapy, occupational therapy, and speech and language therapy are the primary forms of neurorehabilitation currently available at QECH. However, there is not a standardised nor specialised routine for CM patients to access these services, as well as consult with a neurologist, before discharge. One clinician explained that this means CM patients might not receive needed neurorehabilitative care before discharge, preventing the early initiation of rehabilitation. Follow-up assessments for CM patients occur at the hospital, and families are reimbursed for transport. When attending follow-ups on the PRW, the clinical care team determines if any sequelae have arisen from CM and, if so, which sequelae have arisen, subsequently referring the patient to the appropriate specialty (e.g. neurology, physiotherapy) for follow-up care. Patients and caregivers who do not return for follow-up are called to reschedule the follow-up in-hospital or in their village, at which point a mobile clinical care team will travel to the patient’s village to perform follow-up assessments.

### Gaps in caregiver education and knowledge about CM

Caregivers lack important information about the risk factors, symptoms, prevention of CM and its complications, and post-CM care practices. In-hospital healthcare workers reported that caregivers are often discharged with little information about their child’s illness, including its cause and long-term effects, and caregivers of physically disabled children discussed that they do not know how to support their children at home. Some caregivers, however, did describe parenting changes following their child’s discharge from the hospital. These changes included engaging the child in household chores, speaking to or calling a child who cannot talk or hear, engaging the child in light physical exercise, using a torch to determine whether the child can see, and cooking balanced meals. Many caregivers also started to use bed nets in their children’s rooms to prevent malaria. Caregivers stated that these changes were largely guided by advice they received at discharge from their child’s clinical care team.

Healthcare providers described situations and concerns regarding a lack of communication between the medical team and rehabilitation staff regarding patient discharge procedures, post-discharge neurorehabilitation, and medical advice. The absence of standardised discharge and follow-up plans for CM patients has resulted in miscommunications between the medical team and patients’ families. One rehabilitation officer suggested that a standardised discharge plan would enable caregivers to be better educated about the effects of CM on their children. Further, a nurse suggested that CM education take place in the community rather than in-hospital, urging that the government assist by educating community health workers about CM so that community health workers can transfer knowledge to caregivers. It is important to note that some in-hospital healthcare workers and CRWs reported to have given detailed advice to caregivers of CM survivors regarding childcare post-hospital discharge. Caregivers acknowledged receipt of this advice while at the hospital and described that they have faith and trust in the medical providers treating their children at the hospital.

### Caregiver fear of CM recurrence

Caregivers remained fearful of the recurrence of CM in their children and how they would care for their child if he/she fell sick again. Additionally, caregivers expressed worry that their child would never fully recover to how he/she was before falling ill with CM.

### Inability to fund rehabilitative infrastructure

There is a lack of funding to administer free medical equipment, including cerebral palsy (CP) chairs and wheelchairs, to patients in need of it for daily use. For healthcare staff, limited training exists to specialise in rehabilitation, particularly neurorehabilitation, and there are limited opportunities for work due to a dearth of rehabilitation service infrastructure. This has resulted in a lack of expertise to deal with behaviour-related sequelae, as available programmes primarily focus on physical impairments. The lack of rehabilitation staff creates a barrier to provide adequate care for CM survivors with neurological sequelae, and the barrier to access rehabilitative care becomes particularly prominent in communities outside the city centre lacking CRWs. Caregivers expressed difficulty in accessing services centralised in the city due to inability to fund transport to the city hospital. There were previously three organisations in Blantyre City providing small-scale community-based rehabilitation services, all of which were discontinued due to lack of funding.

### Disability stigma and sociocultural barriers to accessing neurorehabilitative care

For post-CM patients with disabilities, social isolation is a barrier for patients and their families that prevents engagement in community activities. Caregivers discussed stigma, including community shame, beliefs about their post-CM child being bewitched, and others mocking the post-CM child. A clinical officer noted the reality of disability stigma in classrooms, stating that children with special needs are seen as a “burden” for teachers who must manage large classes. The lack of individualised care sets children with special needs, including post-CM children with neurological sequelae, up for failure: these children *“get frustrated, and such kids will just drop out from school” (Clinical Officer 3)*. Furthermore, children with behavioural problems may be forced to drop out of school by school headmasters. Caregivers and healthcare workers expressed that a support group would be beneficial means of social support for caregivers more than post-CM children.

### Challenges to continuing care in the community

The lack of community-based rehabilitation was the strongest theme that emerged from participants. Caregivers noted that they do not know of any CBOs providing education and support for families with post-CM children and stated that these organisations should exist. CRWs described a lack of follow-up within the home and community, stressing the importance of community-based therapy. Care does not often continue in the community following hospital discharge, which poses as a barrier to patient recovery. Lack of access to or funding for transportation prevents patients from receiving follow-up care, including attaining neurorehabilitation services, outside of their community. A rehabilitation officer noted, “*the mothers couldn’t turn up because of issues of transport”* (Rehabilitation Officer 2). Many families, especially those living in rural settings, face long distances to health facilities, and unfavourable modes of transportation for physically disabled children. Healthcare workers elaborated that, with lacking social support and difficulties accessing existing follow-up care, caregivers can easily become overwhelmed with taking care of their post-CM child. Clinical officers described that the community, including village chiefs, plays a supportive role in patient recovery: *“there is an impact indeed through involving the chiefs because they have so much power to control the people in the community”* (Clinical Officer 3). Unfortunately, some caregiver experiences were contradictory and emphasised a lack of support in the community. This lack of community-based support extends to the school-setting, where there are no teachers nor programmes available to accommodate post-CM children with special needs. Rehabilitation officers suggested providing special education either within an integrated school or in a separate school for post-CM children: “*I would wish to know if there could be a special school where these children could go”* (Rehabilitation Officer 2).

### Suggestions for implementing community-based rehabilitation

Most participants perceived community-based rehabilitation as a critical component in caring for children who have survived CM and subsequently developed neurodisabilities. Providing incentives to caregivers in the form of food vouchers or transport funding may be efficacious to improve follow-up appointment adherence, especially in instances where attending care in the community is not feasible. Alternatively, some nurses suggested developing community-based palliative care infrastructure for CM patients. Providing a palliative care team with cars to travel to villages for care provision and assessment may mitigate current challenges to providing patients with transportation funds to return to the hospital. Healthcare workers were adamant that more experts be trained in neurodisability management to increase the available labour force when scaling up infrastructure of community-based rehabilitation services. Most urgently, there is a need to gather epidemiological data on neurological disability following CM to inform the building of rehabilitation infrastructure in Malawi and emphasise the breadth of this public health problem.

## Discussion

Neurological sequelae following paediatric CM are a major public health problem in malaria endemic regions. When compared with healthy controls, paediatric CM survivors have a sixty-fold increase of adverse neurological outcomes, including neurodisabilities such as motor impairment, epilepsy, and neurobehavioral sequelae [5, 8]. Over one-third of paediatric CM survivors will develop sequelae; thus, the burden of CM extends beyond mortality to lifetime morbidity [28]. In resource-limited settings, neurodisability results in a substantial burden of disease that impacts the child, family, and community [29]. Consequently, there is a critical need for accessible neurorehabilitation services for post-CM children in Malawi.

This study aimed to identify perceived neurorehabilitation challenges for paediatric CM survivors post-hospital discharge from a specialised unit at QECH in Blantyre, Malawi. We have demonstrated that caregivers lack important knowledge about CM and fear recurrence of CM in their children. Children and families experience substantial stigma and sociocultural barriers to accessing neurorehabilitative care. At a community-level, rehabilitation infrastructure, including trained staff, equipment, and programmes, are extremely limited due to lack of funding. Rehabilitation services are inequitably accessible, and community-based rehabilitation remains largely unavailable.

### Education and knowledge about CM

Previous studies have captured caregivers’ confusion or lack of knowledge regarding their neurodisabled child’s condition and recommended plan of treatment post-discharge [10, 30]. In a past study at QECH, healthcare workers sensed that children could not amply recover until parents understood and accepted their child’s physical disability [10]. Empowering caregivers through increased knowledge and communication about their child’s condition might increase confidence in childcare and help to battle misconceptions and stigma regarding their child [10, 31].

The lack of knowledge regarding CM may be attributed to a lack of education surrounding the illness. Unfortunately, healthcare workers have described their lack of time or expertise to explain details about the child’s condition to caregivers [10]. Nurses and other healthcare workers are busy and usually underpaid, presenting a quality of care issue that poses a barrier to educating caregivers about their child’s illness. Malawi-based qualitative studies on neurodisability have noted that the focus of clinical staff primarily centres on improving CM survival rates than on the long-term disabling effects of the illness [7, 10]. It must be noted that patient caregiver education is a rights-based issue: in Malawi, it is required by law that the primary caregivers of children be educated about their child’s condition and illness; however, caregiver education is often deprioritised in stretched acute medical services [32].

Miscommunications among medical team members can be managed by developing a standard of care for all post-CM children (for those with and without neurodisabilities), including standardised protocols and assessment tools, to encourage the systematic management of post-CM children [10]. These tools should be brief and straightforward to not overload healthcare workers. Additionally, training healthcare workers in counselling could help mitigate miscommunication between healthcare workers and caregivers [10]. Caregivers should be educated to recognise neurological sequelae of CM on discharge and be provided with information regarding where and how to get help for their child if such sequelae appear. To adequately educate caregivers, healthcare workers must communicate in accordance with caregiver knowledge, ability, and preferred learning method to adequately empower and inform caregivers [33]. Making these changes will be a challenge, as they require additional time, training, and evaluative measures.

### Infrastructure funding issues

Healthcare workers in our study discussed the lack of training available to specialise in managing neurodisabilities. Healthcare workers have previously described funding- and opportunity-based barriers to receiving specialised training in paediatric neurodisability; other healthcare workers have suggested that the lack of training is not funding-based but rather due to the lack of motivation and leadership to organise specialised training [10]. Additionally, data from QECH has described healthcare workers as having more knowledge and skills than they realised but lacking the confidence to apply these skills [10]. In resource-limited settings, training staff can be complicated due to inadequate health systems and a lack of specialists; community-based services led by non-specialists may be helpful to increase access to post-CM neurorehabilitation. Caregivers can also be trained to provide basic rehabilitation, such as physiotherapy, at home.

A lack of essential equipment and skilled health personnel increase the existing burden of poverty [34]. Healthcare workers should also be aware of how to maximise the use of limited available resources. Moreover, the timely use of these resources – intense, early management – is critical to ensure prompt intervention when most needed [10].

Sufficient funding does not often exist to thoroughly address the complexity and cost of most rehabilitation interventions; as such, proposed programs are not built and actualised programs are discontinued. Lacking post-CM rehabilitation infrastructure funding exists in-hospital and out-of-hospital, and there is currently no delineation as to who or what should conjure these funds and have ownership over the issue (e.g. the government). Moreover, it is unclear whether this responsibility is at the level of central or district hospitals or whether it is a community-based issue.

### Stigma and sociocultural barriers

The more disabled – whether physically or mentally – an individual is, the more disadvantage he/she will experience accessing healthcare, education, communication, housing, and social services [35]. While neurodisability poses a substantial physical impact on children and their families, strong cultural beliefs and stigma attached to disability can greatly influence families by impacting response to impairments and approach to childcare [7, 35–37]. Healthcare workers have raised concerns regarding the general impact of childhood disability on family life, including risks of abuse and neglect of the disabled child [10]. In Uganda and Malawi, nearly all children with disabilities report experiencing violence, most commonly bullying and verbal abuse [38]. Some neurological sequelae, including epilepsy and seizure disorders, may lead to a child or family’s exclusion by their community due to the association of these disorders with witchcraft and demons and misconceptions that these disorders are contagious [36, 39]. Disability stigma can negatively affect a child’s social life and participation in daily activities, and this is amplified among post-CM children with behavioural issues [7].

In our study, caregivers voiced that the effects of CM on their child have left their family socially isolated; this can lead to feelings of shame and loneliness [7]. Studies on physical disability in Kenya and Malawi have reported that many families experience substantial challenges when caring for children with disabilities, including social exclusion and indignity [40, 41]. The impact of stigma is particularly relevant in the Malawian context, in which the effects of disability extend beyond the individual and affect the immediate and extended family due to a traditionally communal, interdependent culture [42, 43]. One study reported that disabled children are locked in their house to hide from community ridicule or to create time for the parent to take part in other tasks [7]. Children with musculoskeletal disabilities may also be expelled from their house, fall behind in school, or not be included in home or social activities [40, 44]. Consequently, children with neurodisability are at high risk for further health-related issues, poor quality of life, and socioeconomic dependency [30]. Mbale et al. suggested that stigma and discrimination arise from the emotional and social implications of CM on the family and from balancing childcare with the demands of daily life, financial pressures, and child protection [7].

Some caregivers in our study reported that their post-CM child dropped out from school or were forced to drop out of school by school administrators due to neurological sequelae, including physical disabilities and behavioural issues, that could not be accommodated by the school. Interviewees reported a lack of teachers and programmes to accommodate post-CM children with special needs and a critical need for these services. The need for inclusion and better special needs provision in Malawi has been ratified by the government, and thus, all children should be included in school; however, this law is yet to be materialised [45].

In-hospital, caregivers may experience stigma from healthcare workers and other families [10]. In addition to rehabilitation staff, it may be useful to have social workers involved in the post-discharge care of paediatric CM survivors to address complex social concerns and any issues regarding neglect or abuse [10]. It must be acknowledged that neurological sequelae-related stigma and discrimination pose a barrier to accessing child protection [38].

### Continuation of care in the community

In a previous Malawian qualitative study, mothers reported being seen as primary caregivers with occasional support from their husbands, other family members, and church groups [10]. Caregivers have described the immense stress of caring for children with physical disabilities. Mothers have noted that the care burden is typically placed on their shoulders quite literally, as they must carry around their grown child and are thereby limited in completing daily activities, including household tasks, income-generation, and social engagements [10, 46]. Socioeconomic constraints faced by most families in Malawi leads to the prioritisation of income generation and food security over the close care needed by many post-CM children [46].

In our study, healthcare workers described the negative effects of caring for a post-CM child alongside lacking social support. Healthcare workers have addressed that mothers can burnout, become depressed, and experience suicidal ideation when caring for children with physical disabilities, which may lead to neglect or abuse of the child [10, 47, 48]. One Malawian study reported marriage breakdowns as a consequence of a child’s CM [7]. In response, caregivers have described the need for peer support groups, and healthcare workers have suggested group counselling for caregivers of children with neurodisabilities [7, 10]. These groups could inform and empower caregivers through providing psychosocial support during follow-up visits or community-based rehabilitation services. Group meetings could provide a space for caregivers to voice positive and negative experiences and construct novel mediums for support at the community level [49]. In resource-limited settings, group-based interventions, such as peer support groups, have demonstrated success [50].

The challenges of caring for a post-CM child are amplified by the lack of services and policies in Malawi to support these children, particularly children with sequelae that affect the daily life of the child and family. Community health clinics in resource-limited settings have insufficient infrastructure, including equipment, stable and qualified professionals, and medication, to support follow-up care needed by many post-CM children [34]. Several studies have addressed the lack of disability-inclusive planning and inequitable access to accommodations for disabled children [51–54]. Moreover, as described in our study, rehabilitative services – when available – are often centrally-located in urban settings; for families who cannot access or afford transportation and for children with mobility impairments, these services are difficult to impossible to access [51–53, 55]. The inability to access centrally-located services is prevalent in Malawi, where approximately 85% of the population live in rural areas [56]. Approximately two-thirds of the Malawian population lives in poverty, and rural families experience the highest levels of poverty, worst health outcomes, and most difficulty accessing health services [57].

### Solutions and suggestions

Community-based rehabilitation may be able to serve as an interim measure to ensure equitable access of neurorehabilitation services. Building of this infrastructure should be led by Malawian experts who are deeply knowledgeable about the communities that these services seek to serve; involving the user and provider in the infrastructure-building process is critical to inform the generation and implementation of these programs in a manner sustainable, effective, and respondent to the local environment [10]. To prioritise the development of community-based programmes, their need must be highlighted on Malawian policy agendas, adequate government funding must be obtained, and national-level committees should be developed to translate policy to programme [35]. Moreover, rehabilitative programmes should be developed in accordance with the United Nations Convention of Rights for Persons with Disabilities and the International Classification of Function, Disability, and Health and should take a biopsychosocial approach to holistically address caregiver and CM survivor needs [10, 29, 58].

Community-based services should take a multisectoral approach through which male and female caregivers are empowered to care for their post-CM children, family finances are considered, post-CM children can be independent whilst families pursue income-generating activities, caregiver mental health is considered, and the complexities of family and community relationships are addressed [46]. A community-based computerised attention rehabilitation program – as described in Bangirana et al. 2009 – would not be feasible nor sustainable at the community-level due to prohibitive costs, and community-based rehabilitation would be dependent upon trained rehabilitative personnel with sufficient staffing and equipment in each region of Malawi [12]. Instead, community-based rehabilitation teams could provide home-based assessments, such as basic, home-based physiotherapy and occupational therapy, training caregivers in basic physiotherapy techniques that may benefit their child [10]. Successful programs in resource-limited settings have taken a “training-the-trainers” approach, through which caregivers and families are empowered with key skills and knowledge needed to deliver fundamental care to the post-CM child [59]. Programs have also focused on ameliorating family and community stress, promoting healthy coping strategies and providing caregiver support [59]. It must be noted that any large-scale intervention will likely be expensive, require extensive training and capacity-building, and would be a major challenge to implement.

Since there is a wide range of disabling neurological sequelae that post-CM children can develop, CRWs should receive training on basic medical information for children with a large range of disabilities. Services should include two-way dialogue between providers and families regarding the aims of neurorehabilitation and patient outcomes [60]. Lastly, community-based rehabilitation programmes should longitudinally focus on the family and community rather than solely the post-CM child or specific aspect of the child’s disability [10].

The perceived neurorehabilitation challenges identified by this study may apply to a variety of patients with brain injury, including trauma survivors with residual brain injury and survivors of bacterial or tuberculosis meningitis, viral encephalitis, or birth asphyxia. CM provides an important case study highlighting these general gaps in care in Malawi.

### Strengths and limitations

To our knowledge, this is the first study to qualitatively address the perceived neurorehabilitative challenges of paediatric CM survivors in sub-Saharan Africa, applying perspectives from caregivers and healthcare workers. A methodological strength of this study was gathering the views of a wide range of caregivers, healthcare workers, and rehabilitation specialists. Two qualitative data collection methods – IDIs and FDGs – were employed, which may have improved data triangulation by enabling participants to reflect upon experiences, clarify their ideas, and freely share their thoughts. Male and female caregivers ranged in age and looked after children of different ages with varying severity of post-CM neurological sequelae; caregivers and children lived in rural or urban settings throughout Malawi. We encountered some challenges working in English and Chichewa for qualitative data collection and analysis. We occasionally had difficulty getting points across in Chichewa in IDIs and FGDs, as several English words from the guides had no Chichewa translations. Many interviews took longer than anticipated due to miscommunications and misunderstandings between the interviewer and participant. When the interviewer sensed that the participant’s answer did not correspond to the question asked, the interviewer repeated and retranslated the question, which sometimes resulted the participant becoming frustrated with answering a question repeatedly.

Though a wide variety of caregivers were interviewed and thus a broad range of perspectives was represented, sampling could not capture all perspectives of those who care for paediatric CM patients, and it is challenging to determine selection bias of this group. Since 73.3% (*N* = 11) of interviewed staff worked in-hospital at QECH, selection bias was introduced by how staff were gathered for interviews. Staff were gathered from a limited number of sites in-hospital, which may have limited the diversity of views. Most participants lived in Southern Malawi, which may regionally limit perspectives.

### Future directions and local recommendations

To best inform neurorehabilitative programming, ethnographic fieldwork is needed to more deeply understand how children’s post-CM neurological sequelae manifest within the family and community. Research could focus on one aspect of CM neurorehabilitation, such as movement disorders or memory impairment, and delve into precise topics surrounding specific CM outcomes and specific interventions. For example, home-based assessments and interventions, such as safe feeding and basic, home-based physiotherapy performed by caregivers, should be prioritised. According to the WHO Report on Disability, further study is needed to describe barriers faced and unmet needs for neurorehabilitation services [61]. With the findings of this study, we will develop and pilot educational materials for caregivers to increase knowledge regarding CM, neurological sequelae, social implications, and disability rights. We will develop set care guidelines to diminish incidence of miscommunications among medical team members and rehabilitation staff.

## Conclusions

In Malawi, there is a paucity of neurorehabilitation in practice nor clear guidelines to inform rehabilitation in the post-CM period. This study identified perceptions of neurorehabilitation challenges for paediatric CM survivors post-hospital discharge. We found that caregivers lack important knowledge about CM and fear recurrence of the illness in their children. Further, post-CM children and families experience substantial stigma and sociocultural barriers to integrating into their community and accessing neurorehabilitative care. At a community-level, rehabilitation infrastructure, including trained staff, equipment, and programmes, is extremely limited. Rehabilitation services are inequitably accessible, and community-based rehabilitation remains largely unavailable. Additional work is required to expand this study across multiple regions for a holistic understanding of neurorehabilitation needs. There is an urgent need to establish further training of rehabilitation personnel at all levels and to build post-CM rehabilitation infrastructure in Malawi.

## Data Availability

The data analysed in this study are available from the corresponding author upon reasonable request.

## Appendix

### Semi-Structured IDI and FGD Guides

#### Guide for Semi-Structured Interviews with Healthcare Workers at QECH

1. Tell us about your role in caring for children post-CM?
2. Tell us about the processes of discharge care that patients receive on the malaria research ward?
3. What kind of advice is provided?
4. Tell us about the follow-up information that is provided to patients?
5. Approximately what percentage of patients are followed in clinic?
6. Where do you refer your patients?
7. Tell us about any other services you know for children with disability? How do you know about these services?
8. What are the most common types of disability you see?
9. What are the major needs of the children?
10. What do you see as the major gaps in care of post-CM children at QECH?
11. What are the biggest challenges in managing these children from your point of view?
12. What do you think is most important for these children? Is there anything that stops them from getting the help they need at QECH and in the community?
13. What do you think are the main problems the parents face in looking after a child who has had CM?
14. What do you think the parents/caregivers need to know/learn? If you were a parent in this situation what would you want to know?

#### Guide for Semi-Structured Interviews with Rehabilitation Team at QECH

1. Tell us about processes of rehabilitation care for post-CM children?
2. Tell us about the assessments you perform on children in the malaria research ward?
3. How frequently do you see post-CM children in the hospital before they leave?
4. Tell us about your experiences seeing patients once they have left the hospital?
5. What kind of things do you advise?
6. Where do you refer patients?
7. Tell us of any other services you know for children with disability? How do you know about these services?
8. What is your understanding of the effects of cerebral malaria on the child’s brain?
9. What training have you had in managing children with disabilities?
10. Tell us about any special training in taking care of post-cerebral malaria children?
11. What training would you like to have?
12. Tell us if there are barriers preventing you getting this training?
13. What are the most common types of disability you see?
14. What are the major needs of the children?
15. What do you see as the major gaps in rehabilitation care of post-CM children at QECH?
16. What are the biggest challenges in managing these children from your point of view?
17. What do you think is most important for these children? Is there anything that stops them from getting the help they need at QECH?
18. What do you think are the main problems the parents face in looking after a child who has had CM?
19. What do you think the parents/caregivers need to know/learn? If you were a parent in this situation what would you want to know?

#### Guide for Semi-Structured Interviews with Community Health Workers

1. What are the most common disabilities you see in children?
2. What is your knowledge of how CM affects the brain?
3. Tell us whether you are seeing children who have had CM?
4. What special problems do you see in them?
5. What did your training involve?
6. What special training have you received on how to perform rehab on children with brain injury?
7. What training would you like to have?
8. What barriers are there that prevent you from getting this training?
9. Think about a child who has had CM that you see in the community. What normally happens to these children in the community? What services do they get within the community?
10. Who else help them out besides yourself?
11. Tell us about the assessments you perform?
12. What kind of things do you advise?
13. Tell us of any other services you know for children with disability? How do you know about these services?
14. What are the major needs of the children?
15. What do you see as the major gaps in rehabilitation care of post-CM children in the community?
16. What are the biggest challenges in managing these children from your point of view?
17. What do you think is most important for these children? Is there anything that stops them from getting the help they need in the community?
18. What do you think are the main problems the parents face in looking after a child who has had CM?
19. What do you think the parents/caregivers need to know/learn? If you were a parent in this situation what would you want to know?
20. What do you think you can offer?
21. What do you think would be the most helpful thing that CBR workers or others in the community could do for these children and their families during this recovery/rehabilitation period?

*Post-Interview Debriefing:* At the end of each interview, sum-up and debrief with the interviewee. Ask if there is anything else of importance that the interviewee would like to add. Check if the interviewee has any concerns or questions. Reiterate confidentiality. Give contact details for study team in case the interviewee has any concerns. Thank the interviewee for taking part in the study. Five-minute debrief with research assistant and translator to discuss what went well or poorly, what should be changed for next time, and what were the main issues that came out of the interview. Complete note writing. Ensure audio recordings are collected, labelled, and stored securely.

#### Guide for Focus Group Discussions with Caregivers

**Background:** The researcher will explain to caregivers the purpose of the study and talk about neurological impairments.

1. Tell me about your experiences with giving rehabilitation support at home.
  - *Probes*
    - What do you actually do?
    - What challenges do you face?
    - How did you learn what to do?
2. Can you explain to us how you came to know how to do rehabilitation support activities?
  - *Probes:*
    - Any formal or informal training about the rehabilitation support?
    - Is there consistency in doing these activities and who monitors this?
    - How effective are these activities and how do they monitor change?
    - What materials are available to efficiently do these activities?
3. Can you tell us about any rehabilitation support you know of that is currently being given to children with neurological impairments in the homes and community where you live?
4. Can you tell us the need of doing community-based rehabilitation support for children with neurological impairments?
5. How do you think community-based rehabilitation support can best be actualized?

## Abbreviations

CM: Cerebral malaria
CP: Cerebral palsy
CRW: Community rehabilitation worker
FGD: Focus group discussion
IDI: In-depth interview
NGO: Non-governmental organization
CBO: Community-based organization
PRW: Paediatric Research Ward
QECH: Queen Elizabeth Central Hospital
